# The effect of hygienic care practices given to stroke patients on vital signs of patients: A self‐comparison pre‐experimental study

**DOI:** 10.1111/nicc.13283

**Published:** 2025-02-24

**Authors:** İsmail Tunç, Betül Tosun, Ezgi Dirgar

**Affiliations:** ^1^ Department of Nursing, Faculty of Health Sciences Hasan Kalyoncu University Gaziantep Turkey; ^2^ Faculty of Nursing Hacettepe University Ankara Turkey; ^3^ Department of Midwifery, Faculty of Health Sciences Gaziantep University Gaziantep Turkey

**Keywords:** full bed bath, nursing care, stroke, vital signs, wiping bath

## Abstract

**Background:**

Hygiene and skin care are crucial for stroke patients in intensive care, yet the effects of bathing practices on vital signs in mechanically ventilated stroke patients remain underexplored.

**Aim:**

This study aimed to assess the effects of full bed baths and wiping baths on vital signs in stroke patients.

**Study Design:**

A self‐comparison pre‐experimental study was conducted with 90 stroke patients treated in three intensive care units at a Turkish hospital between 10 January 2021 and 01 January 2022. Patients received either a full bed bath (first measurement day) or a wiping bath (second and third measurement days). Vital signs and arterial blood gas values were measured before and after each bath.

**Results:**

Of the participants, 55.5% were male, with a mean age of 64.2 ± 14.8 years. Significant changes in heart rate, systolic blood pressure, diastolic blood pressure and body temperature were observed after the full bed bath (heart rate: *λ* = 0.156, *F* = 43.940; systolic BP: *λ* = 0.484, *F* = 17.981; diastolic blood pressure: *λ* = 0.835, *F* = 7.216; body temperature: *λ* = 0.97, *F* = 142.92; *p* < .001). Similarly, wiping baths resulted in significant changes (heart rate: *λ* = 0.354, *F* = 34.776; systolic blood pressure: *λ* = 0.384, *F* = 16.372; diastolic blood pressure: *λ* = 0.492, *F* = 17.603; body temperature: *λ* = 0.236, *F* = 176.765; *p* < .001). Arterial blood gas changes were significant after wiping baths on Day three (pH: *t* = 3.351, *p* = .001; PaO_2_: *t* = 2.400, *p* = .018).

**Conclusions:**

Both full and wiping bed baths significantly affect vital signs and arterial blood gases in stroke patients. Nurses should tailor bathing practices to patient needs, continuously monitoring vital signs.

**Relevance to Clinical Practice:**

This study highlights how bathing practices impact vital signs and arterial blood gases in intensive care patients. It emphasizes tailoring interventions to patient needs and preferences, as full baths may suit some, while wiping baths offer advantages, particularly for blood pH and PaO_2_ levels.

**What is known about the topic**

- Hygienic care practices, including bed bathing and wiping baths, are important in patient care and affect vital signs.
- Full bed baths and wiping baths can affect physiological parameters like heart rate, blood pressure, body temperature and hemodynamic parameters.
- The effects of these practices on vital signs in stroke patients on mechanical ventilation have been less studied.

**What this paper adds**

- Demonstrates that both full bed baths and wiping baths have significant effects on vital signs and arterial blood gas values in stroke patients on mechanical ventilation.
- Highlights the differences in impact between full bed baths and wiping baths on diastolic blood pressure and body temperature
- Provides evidence that wiping baths can positively affect arterial blood gas values, including blood pH and partial pressure of oxygen (PaO_2_).

## 1 INTRODUCTION

Defined by the World Health Organization as ‘rapidly developing clinical findings due to focal or global impairment of cerebral functions’,^1^ stroke is a pathological condition in the cerebral vessels that occurs with sudden deterioration of brain functions and is an important factor in disability. After deterioration in brain functions, it becomes difficult for the individual to fulfil his/her own care.^2^, ^3^ Stroke patients need help with basic daily activities such as bathing, eating and drinking, walking, moving, dressing, undressing and excretion. One‐third of stroke survivors are able to carry out their daily tasks with the support of someone else.^4^, ^5^

Hygienic care practices include body cleaning, hair, face, ear and mouth cleaning, foot, hand, perineum cleaning and self‐care. The basic part of this care is bathing. Bathing is an important part of self‐care, although it varies from culture to culture.^6^, ^7^ Hygiene and skin care are part of basic care in stroke patients hospitalized in the ICU.^8^ Intensive care patients are in the risk group in terms of circulatory disorders, nutritional disorders, sensory impairment, contamination on the skin surface, skin trauma due to the use of fixation materials, exposure to antibiotics and invasive interventions, multidrug‐resistant microorganisms, and the effect of hygienic care practices given to these patients on their daily lives is very important.^9^

## 2 BACKGROUND

When the literature is reviewed, studies revealing the importance of fulfilling hygienic care practices in the treatment and care of patients are found. Baths can relieve fatigue, prevent infection, maintain tissue integrity, prevent bad odour, provide calmness, increase blood circulation and remove dirt/sweat.^10^ In addition to cleansing and relaxation, bathing also has effects such as accelerating blood circulation, vitality, vigour and a sense of well‐being. Bathing with hot or warm water accelerates blood flow and allows more blood than normal to flow to the superficial arterioles in the skin. After bathing, the person develops an image of relaxation and reduction in tension.^11^ However, it is known that rhythmic movements to the skin during bathing regulate autonomic nervous system function and consequently keep patients' vital signs (body temperature, heart rate, etc.) within physiological limits.^11^, ^12^ Individuals' personal hygiene practices respond to their psychological needs as well as their physical needs. Fulfilling personal care and hygiene needs are recognized as necessary procedures to improve patients' quality of life, social acceptance and well‐being and are very important as they affect patients' psychology and self‐esteem.^10^, ^11^, ^12^, ^13^

Uğraş et al. have emphasized the importance of controlling the patient's fever with bed bath, which is one of the nonpharmacological treatment methods, as well as pharmacological treatment on physician order. They stated that the warm bed/wipe bath method was most commonly used after the use of ventilators in fever control.^14^ In another study, it was found that the ‘diastolic blood pressure’ value of children on mechanical ventilator (MV) reached the highest value immediately after the bed/wipe bath and the lowest value 30 min after the bed/wipe bath. The ‘body temperature value’ reached the lowest value immediately after the bath and was below the values before the bath 30 min after the bath.^15^ In another study conducted in a paediatric intensive care unit (ICU), it was found that the mean systolic blood pressure and diastolic blood pressure of children were highest before bathing and lowest 30 min after bathing.^16^

All this information indicates that hygienic care practices affect many vital parameters in patient care and are closely related to patients' well‐being.

There is a need to know how bed bathing, which is a basic nursing practice frequently performed by health professionals in inpatient hospitals and especially in ICUs, affects vital signs and to what extent this effect contributes to the patient diagnosed with stroke on mechanical ventilation. When the literature on the subject was examined, no study investigating the effect of bed bath and wiping bath on vital signs in adult patients diagnosed with stroke and on mechanical ventilation was found. Accordingly, this study was planned to determine the effect of full bed bath and wiping bed bath, which are considered among the hygienic care practices given to patients with stroke, on the vital signs of patients.

H_0_: Full bed bath given to stroke patients has no effect on vital signs of patients.
H_1_: Full bed bath given to stroke patients has an effect on vital signs of patients.
H_0_: Wiping bath given to stroke patients has no effect on vital signs of patients.
H_1_: Wiping bath given to stroke patients has an effect on vital signs of patients.
H_0_: There is no difference in the change in vital signs according to the type of bath given to stroke patients (full bed bath and wiping bath).
H_1_: There is a difference in the change in vital signs according to the type of bath given to stroke patients (full bed bath and wiping bath).

## 3 METHODS

### 3.1 Design

This non‐randomized self‐comparison pre‐experimental design study was conducted with 90 patients who were treated in three different ICUs (internal medicine ICU, surgical ICU and stroke ICU) of a training and research hospital located in the Southeastern Anatolia Region of Turkey between 10 January 2021 and 1 January 2022. The pre‐experimental design was chosen because it allows for the evaluation of the effects of hygienic interventions on vital signs within the same patient group, thereby providing an initial exploration of the impact of these practices.

### 3.2 Sample

The population of the study consisted of a total of 130 patients who were treated for stroke in three ICUs between the specified dates. The sample size was calculated using the G.Power 3.1 program. When Cohen's effect size of the change in vital signs was of small magnitude (0.25), it was predicted that 5 (repeated measures) repeated measures would be performed for the comparison of single‐group pre‐ and post‐assessment measurements. With a power of 80% and 95% confidence interval, the sample size was determined as 78. Considering the possibility of 15% patient loss, the study sample was calculated as 90 patients. Patients who died during the data collection phase of the study (*n* = 20), who were discharged before the data were completed (*n* = 6), who were transferred to another hospital (*n* = 6) and who were weaned from the MV (*n* = 8) were not included in the sample. The study was completed with 90 patients and in the post hoc power analysis, the power of the study was 87% when the effect size was calculated as 0.37 and *α* = .05.

#### 3.2.1 Inclusion criteria

- 18 years of age or older
- To have been diagnosed with stroke
- Having upper and lower extremities
- Monitoring and good capillary refill
- Being completely dependent in terms of self‐care
- Being connected to a MV
- Patient/patient relatives fill in and approve the informed consent form

#### 3.2.2 Exclusion criteria

- Having severe burns on the skin
- Open wounds on the skin
- Upper or lower extremity amputation
- Inability to measure blood pressure due to casts, etc.
- Discharge at the time of data collection
- Death during data collection
- Refusal to participate in the research

### 3.3 Data collection

Data were collected by the researcher using the patient information form, vital signs and arterial blood gas record form.

### 3.4 Data collection tool

#### 3.4.1 Sociodemographic and medical characteristics form

It is a 14‐question form created by the researchers as a result of a literature review, in which sociodemographic characteristics such as age, gender, marital status, medical diagnosis, chronic disease status and history of drug use are questioned.^15^, ^17^, ^18^

#### 3.4.2 Vital signs and arterial blood gas record form

It is a form routinely used for patient follow‐up in ICUs; heart rate, systolic blood pressure, diastolic blood pressure, oxygen saturation, body temperature, mechanical ventilation mode, PH value, partial arterial oxygen pressure (PaO_2_) value, partial arterial carbon dioxide pressure (PaCO_2_) value, bicarbonate (HCO_3_^−^) value, oxygen saturation (SpO_2_) value and base excess (BE) value are recorded.

### 3.5 Intervention

In the hospital where the study was conducted, patients admitted to ICUs are routinely given a full bed bath during and every 3 days after their initial hospitalization. In addition, patients' bodies are routinely wiped with patient cleaning cloths every day. The researcher and health care assistants performed full bed baths (first measurement day) on the third day of hospitalization and wiping baths on the fourth and fifth days (second and third measurement days) of the patients admitted to the ICU. The procedure to be performed before bathing was explained to ICU patients using verbal and non‐verbal communication techniques. The identifying information of the patients/relatives who agreed to participate in the study was obtained from them or from their files and recorded in the patient identification form. During each intervention, an assistant health care personnel assisted the researcher in turning the patient, positioning and providing materials. In this regard, the researcher informed the patient care staff about the steps of the full bed bath procedure verbally and by video before starting the study. Environmental regulation (25°C ambient temperature, 55% room airflow) was ensured in the ICU before both bed bath applications. To ensure this, a room thermometer was used to monitor airflow and ambient temperature in the ICU. A screen was drawn around the patient's bed and the patient's privacy was taken care of during the whole procedure. The patient's bed brakes were checked and the bed was brought to the appropriate level, and the bed border on the side of the procedure was lowered. The patient was never left alone during the procedure and safety precautions were taken to prevent the patient from falling. The windows and door of the ICU were kept closed against the air flow that may occur in the room before bathing.

Before the full bed bath, two washing tubs were filled with water at an appropriate temperature (38–44°C) and an appropriate amount (two‐thirds of the washing tub). The temperature of the water was measured by the researcher with a water thermometer. Neutral soap was added to the water in one tub, while only clear water was left in the other tub. The neutral soap/shampoo to be used is non‐drying, non‐irritating, non‐irritating and non‐allergenic. The pH of the skin is 5.5, and the neutral soap/shampoo has the same pH or very close to it. In addition, a scrub cloth consisting of an inner layer of pure cotton wrapped with an outer layer of hydrophilic gauze was used for bathing. The patient's hair was washed, and hands and feet were washed by soaking in a tub full of water. When the bathing process started, the stopwatch was started immediately by a nurse independent of the study. Immediately after the application, the stopwatch was stopped and the duration of the procedure was recorded. On average, it took about 22 ± 0.08 min.

Hygienic wipes were used in the wiping bath. These wipes were routinely used in the clinic according to the clinical protocol. The wipes to be used in the bath were heated in a microwave oven at 900 W for 30 s according to the manufacturer's instructions. After heating, the temperature of the wipes was measured with an infrared thermometer. This temperature was aimed to be between 38 and 44°C. The purpose of heating the wipes in the microwave oven is to use the same temperature wiping product in this bath and to prevent factors that may arise from the temperature difference, because the temperature of the water used in the traditional full bed bath is 38–44°C. A separate cleaning cloth was used for each area. No rinsing was required because of the usage characteristics of the cloths. Hair was not washed in the wiping bath. In both baths, patients were washed/wiped from head to toe and front to back using the same steps: head and neck → trunk → four limbs → perineum → back. When the bathing process started, the stopwatch was started immediately by a nurse independent of the study. Immediately after the procedure, the stopwatch was stopped and the duration of the procedure was recorded. On average, it took about 14 ± 0.41 min.

In the full bed bath method, all routine care measures were the same in both bathing procedures except for washing the hair and soaking the hands and feet in water. When the literature was examined, it was found that the effect of general body bathing wears off after 30 min and general physiological changes (such as increase in blood pressure) occurring in the body due to the bathing process return to normal after 9–16 min.^19^, ^20^ Therefore, vital signs were measured and recorded five times before (fifth minute) and after (fifth minute, 10th minute, 20th minute, 30th minute) bathing. Arterial blood gas values of the patients were monitored by obtaining arterial blood gas data from the patient file before and after wiping and/or full bed bathing (30 min). For venous blood pressure measurement, systolic and diastolic blood pressure were measured manually with a stethoscope over the brachial artery using a suitable arm cuff (16 × 30 cm). For body temperature measurement, a non‐contact thermometer was applied to the glabella point on the forehead of the patients. Oxygen saturation and heart rate were measured with a portable pulse oximeter on the fingertips of the patients for 30–40 s. All measurements were performed with the same devices and the devices were calibrated before starting the study.

### 3.6 Data analysis

All data obtained were evaluated with IBM SPSS for Windows, Version 23.0, with appropriate statistical methods according to the characteristics of the data. The suitability of the data for normal distribution was evaluated with the Shapiro–Wilk test, and because the skewness value was between +1.5 and −1.5, it was assumed that the data were normally distributed and it was decided to use parametric tests. Descriptive statistics were expressed as number, percentage, mean and standard deviation; one‐way repeated measures ANOVA analysis, two‐way repeated measures ANOVA analysis and dependent sample *t*‐test were performed. Post hoc Bonferroni correction test was performed to find the difference between groups. Statistical significance level was accepted as *p* < .05.

### 3.7 Ethical consideration

The study was started after receiving the required permissions from the Non‐Interventional Research Ethics Committee (date: 9 August 2021, decision no.: 2021/095), Faculty of Health Sciences, Hasan Kalyoncu University and from the Chief Physician of the state hospital where the study was implemented. We conducted according to the ethics guidelines set out in the Declaration of Helsinki. All the participants in the study were informed about the study, their written/verbal consent was obtained and they were also informed that they could leave the study at any time.

## 4 RESULTS

### 4.1 Descriptive characteristics of the patients

Of the bedridden patients included in the study, 55.5% were male, 62.2% were 61 years of age or older and the mean age was 64.2 ± 14.8 years. In this study, 68.8% of the patients were illiterate, 37.7% had a weight between 71 and 80 kg, 31.1% had hypertension, 98.9% did not consume alcohol, 60% were non‐smokers and 91.2% did not have a family history of stroke (Table 1).

**TABLE 1:** Descriptive characteristics of the patients (*n* = 90).

Descriptive characteristics		*n*	%
Age (year) 64.2 ± 14.8 (min: 25–max: 94)	18–40	6	6.6
	41–50	12	13.4
	51–60	16	17.8
	>61	56	62.2
Gender	Female	40	44.5
	Male	50	55.5
Marital status	Married	88	97.7
	Single	2	2.3
Education level	Illiterate	62	68.8
	Primary school	15	16.6
	High school	10	11.1
	University	3	3.5
Weight (kg) 79.9 ± 10.4 (min: 63–max: 110)	60–70	16	17.8
	71–80	34	37.7
	81–90	27	30.0
	91–100	9	10.0
	101–110	4	4.5
Chronic disease^a^	None	23	19.3
	Hypertension	37	31.1
	Heart disease	24	20.2
	Diabetes mellitus	26	21.8
	Asthma	9	7.6
Alcohol use	Yes	1	1.1
	No	89	98.9
Smoking	Yes	36	40
	No	54	60
Family history of stroke	Yes	8	8.8
	No	82	91.2

^a^ *n* has been repeated.

### 4.2 Changes in vital signs over time in patients undergoing full bed baths and wiping baths

**FIGURE 1:**
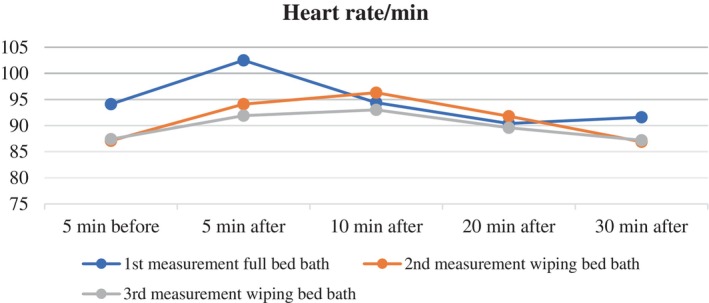
Changes in heart rate (bpm) over time for patients undergoing full bed bath and wiping bath.

When analysing the mean heart rates of patients during the full bed bath on the first day across different time points, the changes in heart rate were found to be statistically significant (Wilks lambda (*λ*) = 0.156, *F* = 43.940, *p* < .001, effect size (*pη* ^2^) = 0.331). When the post hoc Bonferroni correction test was performed to identify the source of the differences, it was found that there was a statistically significant difference between the mean heart rates measured 5 min before the full bed bath and the means measured 5, 20 and 30 min after the bed bath (*p* < .001). The mean heart rate of the patients was highest 5 min after the complete bed bath (Table 2; Figure 1).

**TABLE 2:** Changes in vital signs of patients undergoing full bed and wiping baths over time.

Vital sings	1st measurement day full bed bath		2nd measurement day wiping bath		3rd measurement day wiping bath	
	Mean ± SD	*F*, *p, pη* ^2^	Mean ± SD	*F*, *p*, *pη* ^2^	Mean ± SD	*F*, *p*, *pη* ^2^
Heart rate (bpm/min)						
5 min ago^1^	94.1 ± 22.1	*F* = 43.940 *p* < .001** 2–1 1–4, 5 0.331	87.1 ± 22.6	*F* = 34.776 *p* < .001** 1–2, 3, 4 0.281	87.4 ± 19.8	*F* = 40.964 *p* < .001** 1–2, 3, 4 0.315
5 min later^2^	102.5 ± 22.5		94.1 ± 22.0		91.9 ± 20.3	
10 min later^3^	94.4 ± 22.0		96.3 ± 20.0		93.0 ± 29.7	
20 min later^4^	90.4 ± 19.2		91.8 ± 20.2		89.6 ± 19.7	
30 min later^5^	91.6 ± 20.7		86.9 ± 22.1		87.2 ± 19.8	
Systolic BP (mmHg)						
5 min ago^1^	120.6 ± 14.2	*F* = 17.981 *p* < .001** 1–2, 3 0.168	123.3 ± 17.1	*F* = 16.372 *p* < .001** 3–1 0.155	118.6 ± 15.1	*F* = 8.238 *p* < .001** 2–1 0.085
5 min later^2^	128.5 ± 16.4		130.1 ± 19.2		124.5 ± 16.1	
10 min later^3^	124.8 ± 15.9		133.1 ± 13.4		121.7 ± 21.9	
20 min later^4^	118.9 ± 17.6		123.0 ± 21.9		119.2 ± 19.3	
30 min later^5^	117.5 ± 16.2		119.8 ± 17.4		116.1 ± 15.4	
Diastolic BP (mmHg)						
5 min ago^1^	68.7 ± 9.8	*F* = 7.216 *p* < .001** 2–1 0.075	71.3 ± 8.4	*F* = 17.603 *p* < .001** 2.3–1 0.165	69.3 ± 10.2	*F* = 4.097 *p* < .001** 1–2, 3 0.044
5 min later^2^	72.8 ± 12.5		76.0 ± 14.1		71.8 ± 10.1	
10 min later^3^	70.5 ± 8.8		75.6 ± 7.1		71.9 ± 9.8	
20 min later^4^	68.6 ± 6.0		72.4 ± 6.6		68.7 ± 11.2	
30 min later^5^	69.2 ± 5.3		69.9 ± 6.8		69.0 ± 14.7	
SpO_2_ (%)						
5 min ago^1^	97.80 ± 2.5	*F* = 0.777 *p* = .45 0.009	97.7 ± 2.1	*F* = 1.556 *p* = .216 0.017	97.7 ± 1.7	*F* = 18.830 *p* < .001** 4–1 1–2 0.175
5 min later^2^	96.83 ± 2.5		96.7 ± 2.1		97.1 ± 1.8	
10 min later^3^	97.52 ± 2.1		97.2 ± 1.9		97.5 ± 1.6	
20 min later^4^	98.27 ± 1.9		97.9 ± 1.7		97.8 ± 1.6	
30 min later^5^	97.40 ± 10.5		98.12 ± 1.6		98.2 ± 1.5	
Body temperature (°C)						
5 min ago^1^	36.96 ± 0.43	*F* = 142.928 *p* < .001** 1–2, 3, 4, 5 0.616	37.0 ± 0.40	*F* = 176.765 *p* < .001** 1–2, 3, 4, 5 0.665	36.9 ± 0.42	*F* = 65.511 *p* < .001** 1–2, 3, 4, 5 0.424
5 min later^2^	36.56 ± 0.36		36.6 ± 0.33		36.6 ± 0.34	
10 min later^3^	36.48 ± 0.39		36.6 ± 0.31		36.5 ± 0.36	
20 min later^4^	36.52 ± 0.32		36.6 ± 0.30		36.6 ± 0.34	
30 min later^5^	36.54 ± 0.28		36.6 ± 0.27		36.5 ± 0.43	

*Note*: F = one‐way repeated measures ANOVA. *p < .05. **p < .001 statistically significant. The superscript numbers in denote time points used for statistical comparisons. Significant differences between these time intervals are indicated, highlighting meaningful changes in the measured values.

Abbreviations: BP, blood pressure; Min., minute; *pη* ^2^, partial effect size.

**FIGURE 2:**
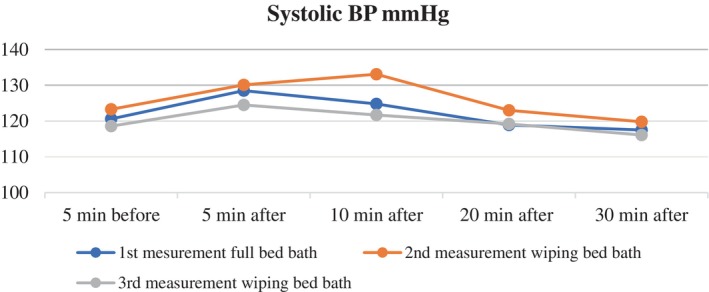
Changes in systolic blood pressure over time for patients undergoing full bed bath and wiping bath.

The change in the mean systolic blood pressure of the patients during the full bed bath was analysed over time, revealing a statistically significant difference (*λ* = 0.484, *F* = 17.981, *p* < .001, effect size (*pη* ^2^) = 0.168). Post hoc Bonferroni tests showed that the mean systolic blood pressure measured 5 min before the bed bath was significantly lower than the means measured 5 and 10 min after the full bed bath (*p* < .001) (Table 2; Figure 2).

**FIGURE 3:**
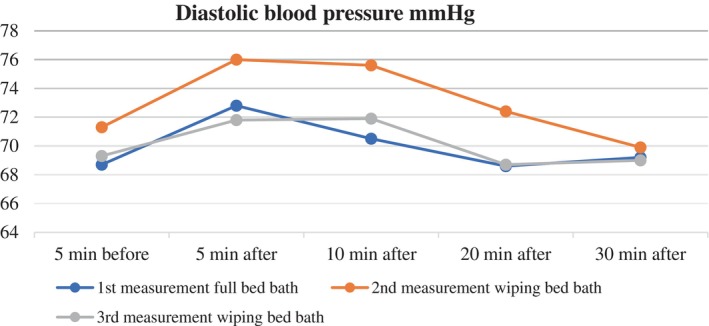
Changes in diastolic blood pressure over time for patients undergoing full bed bath and wiping bath.

When the mean diastolic blood pressures of the patients during the full bed bath were evaluated over time, a statistically significant difference was found (*λ* = 0.835, *F* = 7.216, *p* < .001, effect size (*pη* ^2^) = 0.075). When the post hoc Bonferroni correction test was performed to determine which measurements were significantly different, it was found that there was a statistically significant difference between the mean diastolic blood pressure measured 5 min before and 5 min after the full bed bath (*p* < .001) (Table 2; Figure 3).

**FIGURE 4:**
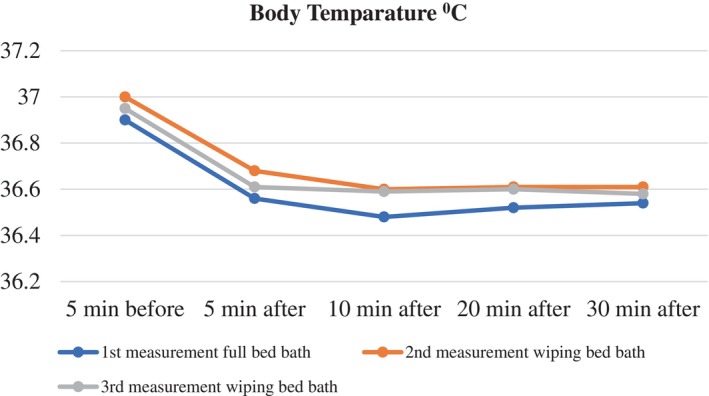
Changes in body temparature over time for patients undergoing full bed bath and wiping bath.

The analysis of mean body temperatures of the patients during the full bed bath showed a statistically significant difference between the five measurements (*λ* = 0.97, *F* = 142.92, *p* < .001, effect size (*pη* ^2^) = 0.616). Advanced statistical tests indicated a significant difference between the mean body temperature measured 5 min before the bed bath and the means measured 5, 10, 20 and 30 min after the bed bath (*p* < .001) (Table 2; Figure 4).

**FIGURE 5:**
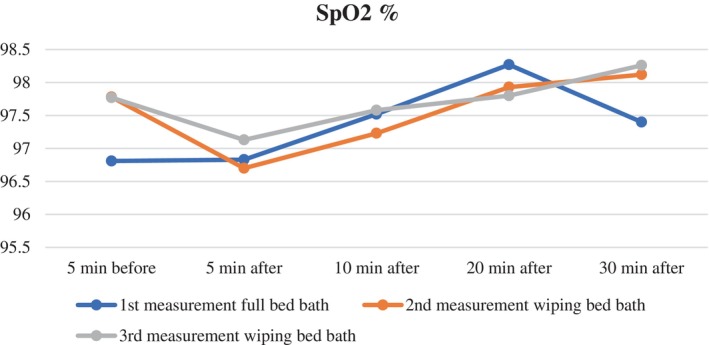
Changes in SpO_2_ over time for patients undergoing full bed bath and wiping bath.

When evaluating the mean oxygen saturation of the patients during the full bed bath, no statistically significant difference was found between the five measurements (*F* = 0.777, *p* = .45) (Table 2; Figure 5).

On the second day, the mean heart rate values of the patients during the wiping bath were analysed, showing a statistically significant difference between the five measurements (*λ* = 0.354, *F* = 34.776, *p* < .001, effect size (*pη* ^2^) = 0.281). The Bonferroni correction test revealed a statistically significant difference between the mean heart rate measured 5 min before the wiping bath and the means measured 5, 10 and 20 min after the wiping bath (*p* < .001) (Table 2; Figure 1).

The comparison of mean systolic blood pressure values of the patients during the wiping bath on the second day showed a statistically significant difference among the five measurements (*λ* = 0.384, *F* = 16.372, *p* < .001, effect size (*pη* ^2^) = 0.155). The Bonferroni correction test found a significant difference between the mean systolic blood pressures measured 5 min before the wiping bath and the mean systolic blood pressures measured 10 min after the wiping bath (*p* < .001) (Table 2; Figure 2).

When evaluating the mean diastolic blood pressures of the patients during the wiping bath on the second day, a statistically significant difference was found among the five measurements (*λ* = 0.492, *F* = 17.603, *p* < .001, effect size (*pη* ^2^) = 0.165). Further analysis showed a significant increase in mean diastolic blood pressure 5 and 10 min after the wiping bath compared with the mean measured 5 min before the bath (*p* < .001) (Table 2; Figure 3).

The mean oxygen saturation of the patients during the wiping bath on the second measurement day did not show a statistically significant difference between the five measurements (*F* = 1.556, *p* = .216) (Table 2; Figure 5).

The analysis of mean body temperatures during the wiping bath on the second day revealed a statistically significant difference among the five measurements (*λ* = 0.236, *F* = 176.765, *p* < .001, effect size (*pη* ^2^) = 0.665). Advanced statistics showed significant differences between the mean body temperatures measured 5 min before and 5, 10, 20 and 30 min after the wiping bath (*p* < .001) (Table 2; Figure 4).

On the third day, the patients were given another wiping bath. The effect of the wiping bath on the patients' vital signs was examined. The analysis of mean heart rate values during the wiping bath on the third day showed a statistically significant difference among the five measurements (*λ* = 0.255, *F* = 40.964, *p* < .001, effect size (*pη* ^2^) = 0.315). Advanced statistical tests found a significant difference between the mean heart rate measured 5 min before the wiping bath and the means measured 5, 10 and 20 min after the bath (*p* < .001) (Table 2; Figure 1).

The comparison of mean systolic blood pressures during the wiping bath on the third day revealed a statistically significant difference among the five measurements (*λ* = 0.406, *F* = 8.238, *p* < .001, effect size (*pη* ^2^) = 0.085). Advanced statistics indicated a significant difference between the mean systolic blood pressure measured 5 min before and 5 min after the wiping bath (*p* < .001) (Table 2; Figure 2).

When analysing the changes in mean diastolic blood pressures during the wiping bath on the third day, a statistically significant difference was found among the five measurements (*λ* = 0.806, *F* = 4.097, *p* < .001, effect size (*pη* ^2^) = 0.044). Further analysis revealed a significant difference between the mean diastolic blood pressures measured 5 min before and 5 and 10 min after the wiping bath (*p* = .01) (Table 2; Figure 3).

The analysis of mean oxygen saturation values during the wiping bath on the third day showed a statistically significant difference over time (*λ* = 0.554, *F* = 18.830, *p* < .001, effect size (*pη* ^2^) = 0.175). Further statistics indicated a significant difference between the mean oxygen saturation measured 5 min before the wiping bath and the means measured 5 and 30 min after the bath (*p* < .001) (Table 2; Figure 5).

Monitoring of the mean body temperatures during the wiping bath on the third day revealed a statistically significant difference among the five measurements (*λ* = 0.134, *F* = 65.511, *p* < .001, effect size (*pη* ^2^) = 0.424). Further statistical analysis showed that the significant difference was between the mean body temperatures measured 5 min before and 5, 10, 20 and 30 min after the wiping bath, with body temperature decreasing after the bath (*p* < .001) (Table 2; Figure 4).

### 4.3 Comparison of mean post‐bath vital signs between wiping baths and full bed bath

In the evaluation of post‐bath measurements according to the type of bath, a statistically significant difference was found in the mean systolic blood pressure (*λ* = 0.371, *F* = 5.955, *p* < .001, effect size (*pη* ^2^) = 0.063). The Bonferroni correction test revealed a significant difference between the wiping bath performed on the second day and the wiping bath performed on the third day (*p* = .03).

When comparing the mean diastolic blood pressures according to the type of bath, a statistically significant difference was found (*λ* = 0.564, *F* = 7.556, *p* < .001, effect size (*pη* ^2^) = 0.078). The Bonferroni correction test showed a significant difference between the full bed bath on the first day and the wiping bath on the second day (*p* < .001).

A statistically significant difference was also found when comparing the mean body temperatures after bathing according to the type of bath (*λ* = 0.371, *F* = 0.920, *p* < .02, effect size (*pη* ^2^) = 0.041). The Bonferroni correction test indicated a significant difference between the full bed bath on the first day and the wiping bath on the second day (*p* < .001) (Table 3).

**TABLE 3:** Comparison of mean post‐bath vital signs between wiping baths and full bed bath.

Bathing type	1st measurement day full bed bath	2nd measurement day wiping bath	3rd measurement day wiping bath	Test (*F*), *p*, *pη* ^2^
Heart rate (bpm/min) Means ± SD	94.63 ± 20.51	91.28 ± 20.74	89.84 ± 19.59	2.796
				0.064
				0.009
Systolic BP (mmHg) Means ± SD	122.0 ± 13.37	125.9 ± 13.88	120.0 ± 15.09	5.955
				<0.001**
				2–3
				0.085
Diastolic BP (mmHg) Means ± SD	70.01 ± 7.01	73.06 ± 7.30	70.19 ± 9.24	7.556
				<0.001**
				1–2
				0.044
SpO_2_ (%)	97.36 ± 3.33	98.22 ± 6.63	97.70 ± 1.47	0.802
				0.385
				0.006
Body temperature (°C)	36.61 ± 0.33	36.71 ± 0.30	36.66 ± 0.34	0.920
				0.002**
				1–2
				0.424

*Note*: F = one‐way repeated measures ANOVA. *p < .05 and **p < .001 are statistically significant.

Abbreviations: BP, blood pressure; *pη* ^2^, partial effect size.

### 4.4 Comparison of arterial blood gas values before and after wiping baths and full bed baths

The data showed that there was no statistically significant difference in mean post‐bath heart rate (*p* = .64) and SpO_2_ (*p* = .38) according to the type of bath. When the comparison of the arterial blood gas values of the patients according to the full bed bath and wiping bath was examined, a statistically significant difference was found between the pH value measured 30 min before and 30 min after the wiping bath on the third day (*t* = 3.351, *p* = .001). In PaO_2_ values, there was a statistically significant difference between the value measured 30 min before and 30 min after the wiping bath on the third day (*t* = 2.400, *p* = .018) (Table 4).

**TABLE 4:** Comparison of blood gas values according to full bed bath and wiping bath.

	1st measurement day full bed bath		95% confidence interval lower/upper bound	2nd measurement day wiping bath		95% confidence interval lower/upper bound	3rd measurement day wiping bath		95% confidence interval lower/upper bound
	Mean ± SD	*t*, *p*		Mean ± SD	*t*, *p*		Mean ± SD	*t*, *p*	
pH									
30 min ago	7.381 ± 0.07	0.645	−0.005/0.011	7.34 ± 0.625	−1.040	−0.201/0.0629	7.41 ± 0.069	3.351	−0.008/0.002
30 min later	7.378 ± 0.06	0.521		7.41 ± 0.075	0.301		7.41 ± 0.071	0.001*	
PaO_2_									
30 min ago	68.9 ± 44.9	1.151	−3.404/12.768	64.2 ± 34.2	−1.767	−4.610/0.270	67.0 ± 38.4	2.400	−6.058/−0.570
30 min later	64.2 ± 26.6	0.253		66.4 ± 30.5	0.081		70.3 ± 35.4	0.018**	
PaCO_2_									
30 min ago	43.7 ± 9.99	−0.381	−1.616/1.096	40.4 ± 9.7	0.803	−0.247/0.583	40.6 ± 11.6	0.889	−0.744/0.284
30 min later	44.0 ± 10.6	0.704		40.3 ± 8.3	0.424		40.8 ± 9.98	0.376	
HCO_3_									
30 min ago	19.5 ± 6.33	−1.225	1.404/5.918	24.8 ± 5.8	1.329	−0.158/0.798	24.5 ± 5.46	0.577	−0.562/1.022
30 min later	21.7 ± 18.0	0.224		24.5 ± 5.3	0.187		24.3 ± 5.78	0.566	
SpO_2_									
30 min ago	95.4 ± 4.51	−1.612	−1.267/0.132	93.7 ± 3.5	−1.404	−44.399/7.626	102.2 ± 91.5	0.876	−10.700/27.591
30 min later	96.0 ± 2.47	0.111		93.6 ± 6.5	0.164		93.8 ± 3.74	0.383	
BE									
30 min ago	1.9 ± 5.8	−1.292	0.072/0.344	0.6 ± 6.1	0.062	−0.415/0.442	0.35 ± 6.59	−0.589	−0.811/0.440
30 min later	1.7 ± 5.3	0.200		0.6 ± 5.3	0.951		0.54 ± 5.98	0.557	

*Note*: *p < .05 and **p < .001 are statistically significant.

Abbreviations: BE, base excess; HCO_3_^−^, bicarbonate value; PaCO_2_, partial arterial carbon dioxide pressure value; PaO_2_, partial arterial oxygen pressure value; SaO_2_, oxygen saturation value.

## 5 DISCUSSION

This study, which found that hygiene practices given to patients diagnosed with stroke had an effect on the arterial blood gas values and vital signs of the patients, and that there was a statistically significant difference between the wiping bath and full bed bath used as hygiene practices, was completed with 90 patients.

In this study, it was found that heart rate, systolic blood pressure and diastolic blood pressure measured 5 and 10 min after the full bed bath and wiping bed bath given to the patients on the first, second and third measurement days increased, while the values started to decrease after 20 min and fell below the first measurement after the 20th minute. The acceleration of blood circulation and increase in heart rate during the cleaning process with hot water during bed bathing is physiologically expressed similarly in the literature.^6^, ^17^ Ketelhut and Ketelhut explained the physiological effect of sauna bathing in their study and explained that the acceleration of circulation and increase in heart rate immediately after the bath is related to the movement and massage during the bath and has an effect similar to physical activity. They then reported that the heart rate, systolic and diastolic pressure decreased with vascular vasodilation caused by relaxation caused by bathing and massage. In this case, it was thought that the bed bath had a desirable effect in terms of blood pressure and heart rate.^21^

It is known that patients lose heat through radiation and conduction when their body temperature is high. Patient exposure to environmental heat loss is common when performing basic body care such as bed bathing.^18^ In our study, when the change in body temperature with respect to time was examined, it was observed that it decreased by approximately 1°C in a similar manner immediately after the full bed bath on the first measurement day and wiping baths on the second and third measurement days, and there was no significant increase when measured again 30 min later. During hygienic practices, in addition to exposing the naked body to the environment, patients are in direct contact with bath water, which can often cool down and increase patients' body temperature. While the positive effects of body bathing can be utilized to bring the body temperature of patients with elevated body temperature to normal limits, it can also cause hypothermia in intensive care patients and reduced circulation and tissue oxygenation.^17^, ^22^ For this reason, nurses should keep the body temperature of patients within normal limits during hygienic care practices, and bath water temperature and room temperature should be under control.^22^

In this study, when the change in oxygen saturation over time before and after the bath applications given to the patients was evaluated, it was found that the saturation generally remained at similar values after each application, but there was a statistically significant difference in oxygen saturation in the measurements made 30 min after the wiping bath on the third day. There is no consensus in the literature on the effects of bed bathing on saturation in critically ill patients. In a study conducted in Egypt, a significant negative correlation was found between bath time and oxygen saturation level in critically ill patients.^23^ Studies have emphasized the negative effect of this practice on the respiratory functions of patients by stating that prolonged bathing practices cause stress, tachypnoea and hypothermia in patients and saturation decreases in critically ill patients.^17^, ^22^, ^24^ Kızıl and Şendir showed that saturation increased after bathing in MV‐dependent children and showed the positive effect of bathing. They stated that the wiping and rubbing process during bathing helps oxygenation and helps the ventilation–perfusion balance of tissues by stimulating blood circulation.^15^ In the study, because all patients were connected to a MV, respiratory rate, respiratory depth and other respiratory symptoms such as dyspnoea following respiration could not be evaluated independently. However, the wiping and scrubbing process may have accelerated circulation and increased oxygenation in the patients and may have increased the saturation of the patients in the measurement made at 30 min after the wiping bath on the third day.

When the arterial blood gas values of the patients in the study group were analysed, it was determined that the mean blood pH value increased significantly after the wiping bath on the third day. In addition, it was determined that the partial pressure of oxygen in the blood (PaO_2_) value of the patients increased significantly after the wiping bath on the third day. This result suggests that this result should be evaluated together with the saturation values of the patients on Day 3. The wiping bath given to the patients together with other parameters affecting respiratory functions in MV‐dependent patients may have positively affected PaO_2_, saturation, tissue oxygenation and blood pH value of the patients. While there is no study in the literature examining the effect of wiping bath on blood pH and PaO_2_, Hatefi et al. showed that whole body massage had a positive effect on saturation, pH and PaO_2_ in patients. It can be concluded that the wiping bath may have the same effect as whole body massage.^25^

In the comparisons made in this study, it was found that traditional full bed full body bath and wiping bath were different in terms of their effects on diastolic blood pressure and body temperature values. In the literature, there are studies investigating the effect of traditional full bed bathing and wiping bathing on vital signs of patients.^17^, ^22^, ^26^, ^27^, ^28^ While some studies emphasize that the two types of bathing do not significantly differ in vital signs,^17^ some studies have argued that the effects of wiping baths on vital signs are more positive.^15^, ^22^, ^29^ This suggests that nurses can use traditional full bed bathing and wiping baths in patients' self‐care practices by considering patient needs. Nurses should keep in mind that these two practices may change the vital and hemodynamic parameters of the patients and should pay attention not only to the application of the technique but also to the behaviour of the patients. It is essential for nurses to provide individualized care to patients with whom they cannot communicate verbally by analysing facial expressions and/or monitoring and continuously monitoring the vital signs of the patients.^17^, ^22^, ^26^, ^27^, ^28^

### 5.1 Limitation

The results of this study are limited to the selected sample of patients treated in ICUs with a diagnosis of stroke and cannot be generalized to the broader population. Additionally, as a pre‐experimental study, the absence of a control group or random assignment introduces potential limitations in internal validity. The results are also influenced by the fact that vital signs and arterial blood gas values can be affected by a variety of factors beyond the hygienic practices assessed. Therefore, any changes observed cannot be solely attributed to the hygienic interventions, as other variables may have contributed to the findings.

### 5.2 Implications and recommendations for practice

The study shows that full bed hygienic care practices significantly affect vital signs and hemodynamic parameters in stroke patients. To maximize the benefits of these practices, it is essential to educate caregivers on the benefits and techniques of both bed baths and wiping baths, ensuring they understand their impact on patients' vital signs and hemodynamic parameters. The results highlight the importance of customizing bathing interventions to meet individual patient needs and preferences. While traditional full bed baths may be suitable for some patients, wiping baths might be more appropriate for others, given their positive effects on vital signs and hemodynamic parameters. Nurses should provide individualized care by observing patients' facial expressions and continuously monitoring vital signs, especially for those who cannot communicate verbally. Additionally, further randomized controlled experimental studies are needed to explore how new techniques and practices in hygienic care can be more effectively utilized for patient benefit.

## 6 CONCLUSION

This study provides empirical evidence that both full bed baths and wiping baths elicit significant alterations in the vital signs and arterial blood gas values of mechanically ventilated stroke patients. Heart rate, systolic and diastolic blood pressures exhibited an immediate post‐bath increase, followed by a progressive decline below baseline measurements within thirty minutes. A consistent post‐bath reduction in body temperature underscores the critical need for meticulous control of environmental and water temperatures during hygienic care interventions. Notably, wiping baths were associated with improvements in arterial blood gas parameters, particularly blood pH and PaO_2_, indicating potential respiratory and hemodynamic benefits in critically ill stroke patients.

The findings underscore the imperative for individualized hygienic care protocols in intensive care units, emphasizing continuous monitoring of physiological parameters to ensure patient safety and optimize clinical outcomes. This study enriches the existing literature on nursing interventions in critical care, advocating for the integration of patient‐specific hygiene practices to enhance care quality for stroke patients. Further randomized controlled trials are warranted to corroborate these findings and explore advanced hygienic care strategies that promote optimal patient outcomes.

## AUTHOR CONTRIBUTIONS

**İsmail Tunç:** conceptualization, data curation, formal analysis, investigation, software, writing — original draft. **Betül Tosun:** conceptualization, data curation, formal analysis, investigation, methodology, software, writing — original draft, supervision, review and editing. **Ezgi Dirgar:** conceptualization, supervision, writing — review and editing.

## Data Availability

The data that support the findings of this study are available from the corresponding author upon reasonable request.
